# Genome-wide scans for the identification of *Plasmodium vivax* genes under positive selection

**DOI:** 10.1186/s12936-017-1882-0

**Published:** 2017-06-06

**Authors:** Hai-Mo Shen, Shen-Bo Chen, Yue Wang, Bin Xu, Eniola Michael Abe, Jun-Hu Chen

**Affiliations:** 1 National Institute of Parasitic Diseases, Chinese Center for Disease Control and Prevention, WHO Collaborating Centre for Tropical Diseases, National Center for International Research on Tropical Diseases, Key Laboratory of Parasite and Vector Biology Ministry of Health, 207 Rui Jin Er Road, Shanghai, 200025 People’s Republic of China; 2 Institute of Parasitic Diseases, Zhejiang Academy of Medical Sciences, Hangzhou, 310013 People’s Republic of China

**Keywords:** *Plasmodium vivax*, Haplotype-based detecting, Positive selection, Invasion, Immune evasion, Drug resistance

## Abstract

**Background:**

The current trend of *Plasmodium vivax* cases imported from Southeast Asia into China has sharply increased recently, especially from the China–Myanmar border (CMB) area. High recombination rates of *P. vivax* populations associated with varied transmission intensity might cause distinct local selective pressures. The information on the genetic variability of *P. vivax* in this area is scant. Hence, this study assessed the genetic diversity of *P. vivax* genome sequence in CMB area and aimed to provide information on the positive selection of new gene loci.

**Results:**

This study reports a genome-wide survey of *P. vivax* in CMB area, using blood samples from local patients to identify population-specific selective processes. The result showed that considerable genetic diversity and mean pair-wise divergence among the sequenced *P. vivax* isolates were higher in some important gene families. Using the standardized integrated haplotype score (|iHS|) for all SNPs in chromosomal regions with SNPs above the top 1% distribution, it was observed that the top score locus involved 356 genes and most of them are associated with red blood cell invasion and immune evasion. The XP-EHH test was also applied and some important genes associated with anti-malarial drug resistance were observed in high positive scores list. This result suggests that *P. vivax* in CMB area is facing more pressure to survive than any other region and this has led to the strong positive selection of genes that are associated with host-parasite interactions.

**Conclusions:**

This study suggests that greater genetic diversity in *P. vivax* from CMB area and positive selection signals in invasion and drug resistance genes are consistent with the history of drug use during malaria elimination programme in CMB area. Furthermore, this result also demonstrates that haplotype-based detecting selection can assist the genome-wide methods to identify the determinants of *P. vivax* diversity.

**Electronic supplementary material:**

The online version of this article (doi:10.1186/s12936-017-1882-0) contains supplementary material, which is available to authorized users.

## Background

The Greater Mekong Sub-region is one of the most threatened foci of malaria in Southeast Asia [1, 2] because it accounts for more than half of the malaria cases reported in the region and an estimated 75% of malaria deaths occurred in Myanmar [3]. Moreover, China and Myanmar border region have the highest malaria incidence among the international border regions in Asia [1, 4]. Case management of imported malaria within the context of malaria pre-elimination is increasingly considered to be relevant because of the risk of resurgence [5, 6]. The genetic diversity and evolutionary plasticity of *Plasmodium vivax* constitute major obstacles for malaria elimination. Several *P. vivax* isolates genome sequencing projects were completed recently [7, 8], but information is scant about the genetic variability in China–Myanmar border (CMB) area. The lack of reliable and adequate information on the genetic variability of *P. vivax* genome creates knowledge gap and uncertainty about the preventive strategies for effective malaria control.

The development in recent years has transformed *Plasmodium* genome sequencing from a complicated task into a well-defined set of procedures and pipelines that are potentially accessible to all researchers [9]. These pipelines provide a deep understanding on the intricacies of parasite population, generate reliable information to enhance monitoring their effects and to raise alert for action in case of emergency [10]. Though, several methods are available but haplotype-based approaches are particularly useful for identifying variants that have undergone a partial or incomplete selective sweep by using metrics that probe such reduced haplotype diversity [11, 12]. The selective sweep results in the rapid rise in frequency of beneficial alleles accompanied by a reduction in haplotype diversity in the neighbourhood of functional mutations due to a hitching effect.

Previous studies on *Plasmodium falciparum* adapted the haplotype-based XP-EHH (cross-population extended haplotype homozygosity) test on parasites from Senegal and detected that several loci are associated with drug resistance genes, including some known signals at chloroquine resistance transporter gene (*crt*), bifunctional dihydrofolate reductase gene (*dhfr*) and multidrug resistance protein 1 gene (*mdr1*) [13]. Mobegi et al. reported a genome-wide survey in Guinea for positive selection from the standardized integrated haplotype test and identified ten chromosomal loci that had two or more SNPs with high |iHS| score (top 1% of the distribution). It revealed strong signatures around the two major chloroquine resistance genes (*crt* and *mdr1*) and weak signatures around the sulfadoxine resistance gene (*dhps*) [14]. Similar methods were applied to analyse the genomes of *P. vivax* parasites collected from patients in CMB area and the positive selection signatures on genes associated with invasion, immune evasion and drug resistance were observed. These results showed that the genetic diversity is similar to the global scale even in such small area. The mean pairwise divergence among the sequenced *P. vivax* isolates is higher in gene families associated with red blood cell invasion and immune evasion. This suggests that *P. vivax* in CMB area is facing more pressure to survive than any other region.

It is important to identify molecular markers of drug resistance for improvement on drug resistance surveillance and prevention of complications that arises from inadequate therapies to be achieved. Therefore, this study identified sign of hard selective sweep involved in drug resistance genes that are solely consistent with the history of drug use during the national malaria elimination programme in CMB area. The identification of these signatures of positive selection could help us to identify drug resistance genes as well as new vaccine candidates.

## Methods

### Ethics statement

The study was approved by the Ethics Committee of the National Institute of Parasitic Diseases (NIPD), China CDC. The study protocol, potential risks and potential benefits were explained to the participants. After agreement by the participants to be recruited into the study, the informed consent to participate was given and all the participants provided written informed consent.

### Collection of genomic data

Genome data previously published from seven monkey adapted strains: Sal I [15], Belem [16], Chesson [17], Brazil-I, India-VII, North Korean, Peru [18] and Mauritania-I [19] were used for the analyses. Meanwhile, six human clinical isolates genome were referenced: Cambodia (C08, C15, and C127) and Madagascar (M08, M15, M19) [20]. Raw sequences of the strains deposited in the GenBank database under the following SRR number were downloaded, these include; (Sal I resequencing: SRR575089, Madagascar: SRR570031, SRR828416, SRR572651, Cambodia: SRR572648, SRR572650, SRR572649, Brazil: SRR332573, SRR332569, IQ07: SRR064844, SRR073125, India VII: SRR332913, SRR332914, North Korea: SRR332565, SRR332562, Mauritania I: SRR332413, SRR332408, Belem: SRR575087 and Chesson: SRR828528). More so, this study employed recently released genotype calls data set (Variant Call Format file) with several countries throughout the world: Cambodia, China, India, Indonesia, Laos, Malaysia, Myanmar, Thailand, Vietnam and Papua New Guinea [21]. The SNP information and allele frequencies were downloaded from the *P. vivax* Genome Variation Project [8]. In addition, the annotation of the Sal I reference from PlasmoDB database was downloaded [22].

### Sampling *Plasmodium vivax* parasites from malaria patients and genome sequencing

The blood sample were collected from six clinical malaria cases that were microscopically positive for *P. vivax* with high parasite density (40,000–260,000 parasites/μl) from Tengchong county, an area of China–Myanmar border of Yunnan province in 2010. The samples were confirmed *P. vivax* mono-species infection by *Plasmodium* species PCR-based diagnosis [6]. Genomic DNA was extracted from each frozen blood sample using the QIAGEN DNeasy Blood & Tissue Kit (Qiagen, UK), and sheared into 500 bp fragments to construct the Illumina sequencing libraries with insert sizes of 250 bp. Previously, an initial sequencing result of sample CMB-1 and subsequent analysis was reported [23]. Using the same method, all libraries on Illumina HiSeq2000 were sequenced and generated an average of 120 M paired-end reads of 125 bp. All Illumina raw sequencing reads have been submitted to the NCBI Short Read Archive (BioProject no. PRJNA284437). All reads were filtered by removing the adapter sequences and low quality sequences with Trimmomatic-3.0 [24].

### Identification of SNPs from *Plasmodium vivax* parasites

Sequencing reads from 25 samples (6 samples from CMB area and 19 reference samples) were mapped to *P. vivax* Sal I genome using BWA [25] with default parameters and SNPs called using SAMTOOLS [26]. High-quality single nucleotide polymorphism (SNPs) that met the following criteria were obtained: (1) quality scores >30; (2) not at the extremes of the genomic coverage distribution (<5- or >1000-fold), which normally reflect deletions or copy number variants. Due to the variety of quality in the reference and the CMB samples, the SNPs on 14 chromosomes were retained and the rest was excluded. SNPs were also excluded from analysis if they were positioned within sub-telomeric regions or the repetitive sequences. A total of 188,757 SNPs remained for analysis after filtering. Then, the distribution of high-quality SNPs in each gene was calculated using an in-house Perl script, and a principal component analysis (PCA) of all strains was performed using all the SNPs identified. The majority allele within each infection was identified for use in the analysis of population allele frequencies. In the subsequent analysis, the SNP dataset was further filtered to exclude samples with missing calls at >5% of all positions.

### Positive selection tests

For the high-quality SNPs in the CMB area population, the nucleotide diversity ($\hat{\pi }$) and the Watterson’s estimator ($\hat{\theta }$_ω_) were estimated for the whole genome mutation rate in 2 kb slide windows across each chromosome in ARLEQUIN-Ver3.5 [27]. Integrated haplotype score (iHS) and cross-population extended haplotype homozygosity (XP-EHH) in Selscan-Ver1.10a were used to detect signals of recent or ongoing positive selection [28]. These statistical analyses are based on the selective sweep model, where a mutation arises on a haplotype that quickly sweeps toward fixation and reduces diversity around the locus.

iHS is the standardized log ratio of the integrated extended-haplotype homozygosity (EHH) [29], which calculated the six CMB clinical samples by tracking the decay of haplotype homozygosity for both the ancestral and derived haplotypes extending from every SNP site [30]. SNPs with inferred ancestral states and a minor allele frequency of at least 5% were used for iHS. Each unstandardized scores were normalized in frequency bins across the entire genome. During the EHH computation of each SNP loci, if the start/end of a chromosome arm is reached before EHH < 0.05 or if a physical distance (kbp) between two markers >200 is encountered, the calculation is aborted. XP-EHH is the standardized log ratio of the integrated site-specific EHH at core SNPs between populations A and B, which is defined in this study as CMB samples and the reference samples from all over the world respectively [11]. Site-specific EHH does not require markers to be polymorphic within the population. Therefore, it can detect selective sweeps for alleles that have risen to fixation. In this calculation, the sums in each locus were truncated at the SNP with EHH value <0.05 or if the computation extends more than 1 Mbp from the core loci. Previous analyses suggested that iHS has maximum capacity to detect selective sweeps that have reached moderate frequency, while XP-EHH has the capacity to detect selective sweeps at high frequency, thus making the two tests complementary.

## Results

### Whole genome sequencing of *P. vivax* parasites and mapping

Previously, a direct sequencing approach which requires only high parasitemia for *P. vivax* sample without leukocytes filtration was reported. The same method was used to sequence the clinical isolates of *P. vivax* genome sequence obtained in the China–Myanmar border area. Among hundreds of samples collected from CMB area, only six sequenced results were good enough for the standard and provided enough coverage so far. This study generated between 34 and 215 million paired-end reads with an average read length of 125 bp from each of these samples (Table 1).

**Table 1:** Sequencing and mapping summary statistics of six samples from CMB area

Samples	Pv96	Pv113	Pv128	Pv129	Pv138	Pv204
Sequencing and mapping						
Number of reads	34,817,405	215,617,516	36,303,847	71,449,001	36,731,899	34,210,120
Mapped on *P. vivax*	5,316,739	32,851,379	4,816,957	8,108,382	5,962,674	10,159,389
Mapped (%)	15.27	15.24	13.27	11.35	16.23	29.7
Mean mapping quality	35.38	37.63	37.01	36.72	38.28	36.05
Coverage						
Coverage fold	17.32	108.72	14.55	21.16	19.79	40.41
Genome covered (%)	93.28	97.32	93.06	91.24	95.96	94.85
Chromosomes covered (%)	98.50	99.58	97.29	95.49	99.02	99.24

Sequence reads from the 6 CMB samples and 19 reference isolates were aligned to the *P. vivax* Sal I reference genome. A variable proportion of reads (11–29%) from all the isolate samples were mapped to the reference. High-quality consensus base calls for an average 94.28% coverage on whole genome and 98.18% on chromosomes were generated for each sample. Despite the presence of some poorly covered regions, the mapped reads allows for comparison between multiple individuals for the 14 chromosomes of the *P. vivax* genome. Of the 188,757 SNPs, 6 CMB samples involved a total of 109,665 SNPs. Excluding the low-frequency SNPs in each population (55,301 SNPs with minor allele frequency <5%), a total of 133,456 SNPs across the 25 isolates were identified.

**Fig. 1:**
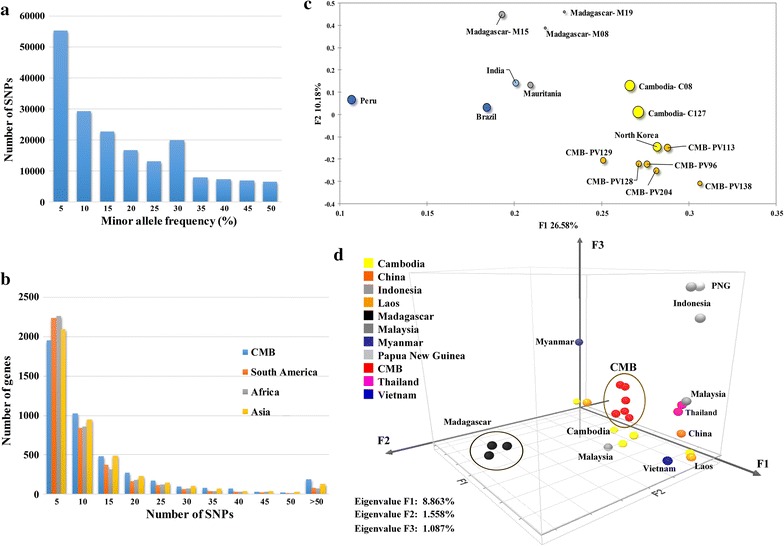
Summary of the SNPs frequency distribution and PCA shows the regional differences. **a** Frequency distribution of the minor alleles for each of the SNPs scored in a population sample of 6 *P. vivax* clinical isolates from CMB area. **b** Distribution numbers of genes (N = 4653 analysed in total) with each given number of SNPs in different population. **c** Principal component analysis based on 133,456 SNPs in CMB isolates and reference strains. The samples are dyed by their geographic origin: *blue* for Peru and Brazil from South America, *green* for India from South Asia and *grey* for Madagascar and Mauritania from Africa, and *yellow* for Cambodia, North Korean and CMB. The PCA result shows that *P. vivax* clustered according to their geographic origin. **d** Three-eigenvector PCA using variation data from CMB isolates and some Asian reference strains. The clinical isolates outside Asia were used as outgroup indicators: Papua New Guinea (PN0046-C), Indonesia (PJ0007-C and PJ0009-C) and Madagascar (M08, M15 and M19). Each isolate is dyed according to its origin and the percentage contribution of each eigenvector is indicated

Single nucleotide polymorphism were identified across the 4653 genes on 14 chromosomes in CMB samples and 2467 genes with more than 5 SNPs. These genes were considered informative for comparisons of polymorphic nucleotide site frequency spectra for such a comprehensive analyses. CMB samples were observed to have genes with higher SNPs than samples from other parts of the globe in each cluster. This suggests a greater genetic diversity and faster evolution in CMB area (Fig. 1a, b).

Principal component analysis (PCA) of all strains was performed to assess the regional differences. As part of the Asia isolates, the CMB isolates illustrated a higher discrepancies with the Sal I genome (Fig. 1c). PCA result shows that *P. vivax* clustered generally according to their geographic origin and the host switch in reference was not a major determinant of the genetic diversity. As previously reported [31], the India strain was tagged under the Africa category instead of Asia, and more closely to the South America clades in PCA approach. Another PCA test was performed to explore the Asian population structure of *P. vivax* (Fig. 1d). The major axis of differentiation clustered the CMB samples together, and divides them from China, Thailand and Vietnam samples. The second and third principal components define a distinct outgroup Madagascar and Papua New Guinea clusters, respectively. It is also important to note that some samples from the same area (Cambodia and Laos) with different periods were also divided in the major axis, suggesting higher diversity from other Asia populations.

### Haplotype-based detecting positive selection

**Fig. 2:**
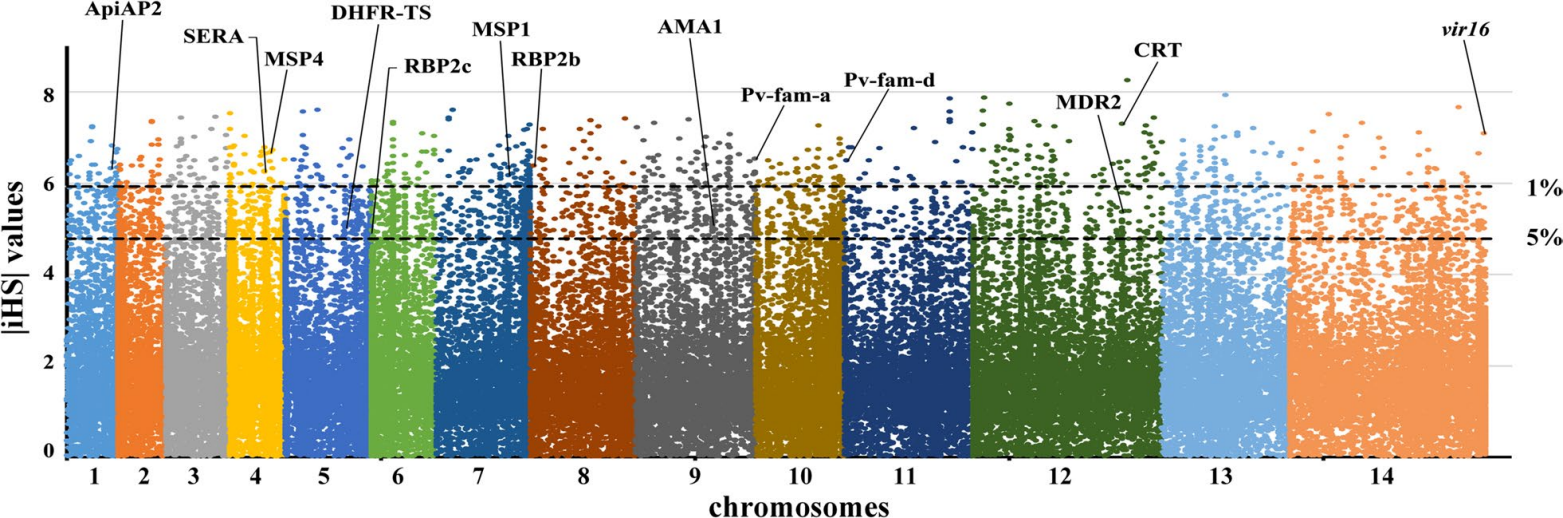
Genome-wide scan of standardized |iHS| for *P. vivax* SNPs with minor allele frequency of at least 5% in CMB area. Individual chromosomes are identified by alternate colouring of their SNPs, with high scoring SNPs highlighted, indicating loci most likely to have been under recent positive directional selection

This study used integrated haplotype score (iHS) statistic to detect incomplete sweeps and cross-population extended haplotype homozygosity (XP-EHH) in case the sweep is near fixation within population. The two methods are complementary in terms of their scope. Using standardized integrated haplotype score (|iHS|) for all SNPs (MAF > 5%) in the CMB samples, all 14 chromosomal regions were identified with SNPs above the top 5% value of the randomly expected distribution (|iHS| > 4.78), especially the top 1% (|iHS| > 5.93) (see Additional file 1: Table S1 for a complete list of the top 5% score genes). The top 1% SNP locus include 356 gene encoding proteins, most of their families are associated with red blood cell invasion and immune evasion such as merozoite surface protein 1 (MSP1, |iHS| = 6.15), MSP3.1 (|iHS| = 6.35), MSP4 (|iHS| = 6.65), MSP1P (|iHS| = 5.96), tryptophan-rich antigen (PVX_097575, |iHS| = 7.00), rhoptry neck protein 2 (RON2, |iHS| = 6.41), serine-repeat antigen 4 (SERA4, |iHS| = 6.77), merozoite adhesive erythrocytic binding protein (MAEBL, |iHS| = 6.18), reticulocyte binding protein 2b (RBP2b, |iHS| = 6.37), reticulocyte binding protein 2c (RBP2c, |iHS| = 5.80) (Table 2), as well as VIR family, such as variable surface protein 4 (Vir4, |iHS| = 7.31) (Fig. 2). In addition, most of the gene family members shown up in the top 5% list are located close to each other on the chromosome. For example, eight SERA genes are contiguously arranged on chromosome 4, gene PVX_003845 (*sera4*) involved the top 1% SNP locus (|iHS| = 6.77), others were also in the 5% iHS candidate list. This result is expected because the process of positive natural selection increases the prevalence of both selected variant as well as of nearby variants, generating local regions of extended haplotypes. Elevated |iHS| values were observed in some important individual gene encoding proteins, such as apical membrane antigen 1 (AMA1, |iHS| = 5.15), cysteine-rich protective antigen (CyRPA, |iHS| = 5.23), GPI-anchored micronemal antigen (GAMA, |iHS| = 4.94), and reticulocyte binding protein 2a (RBP2a, |iHS| = 4.79). The positive selection result is similar to results obtained from previous studies [32, 33], and the selection of vaccines targeting polymorphic antigens may explain the hurdle in eliciting cross-protective immune responses.

**Table 2:** Notable *P. vivax* genes with their associated positive selection statistics

Gene and product description	|iHS|	XP-EHH	$\hat{\pi }$
Mosquito transmission			
PVX_095475, circumsporozoite and TRAP-related (CTRP)	4.9995		0.0053
PVX_118360, TRAP-like protein (TREP)	6.1642		0.0011
Red blood cell invasion			
PVX_099980, merozoite surface protein 1 (MSP1)	6.1512		0.0185
PVX_099975, merozoite surface protein 1 paralog (MSP1P)	5.9582	1.9937	0.0036
PVX_092975, merozoite adhesive erythrocytic binding (MAEBL)	6.1795		0.0024
PVX_097575, tryptophan-rich antigen	6.9579		0.0082
PVX_092275, apical membrane antigen 1 (AMA1)	5.1501		0.0056
PVX_117880, rhoptry neck protein 2 (RON2)	6.4102		0.0027
PVX_003845, serine-repeat antigen 4 (SERA4)	6.7713		0.2834
PVX_121920, reticulocyte binding protein 2a (RBP2a)	4.7869	1.9589	0.0004
PVX_081270, reticulocyte binding protein 2b (RBP2b)	6.3732		0.0036
PVX_090325, reticulocyte binding protein 2c (RBP2c)	5.7962		0.0353
Drug resistance			
PVX_087980, chloroquine resistance transporter (CRT)	4.9064		0.0024
PVX_118062, chloroquine resistance marker protein	7.2833	1.8212	0.0119
PVX_118100, multidrug resistance protein 2 (MDR2)	5.3704	1.8858	0.0093

**Fig. 3:**
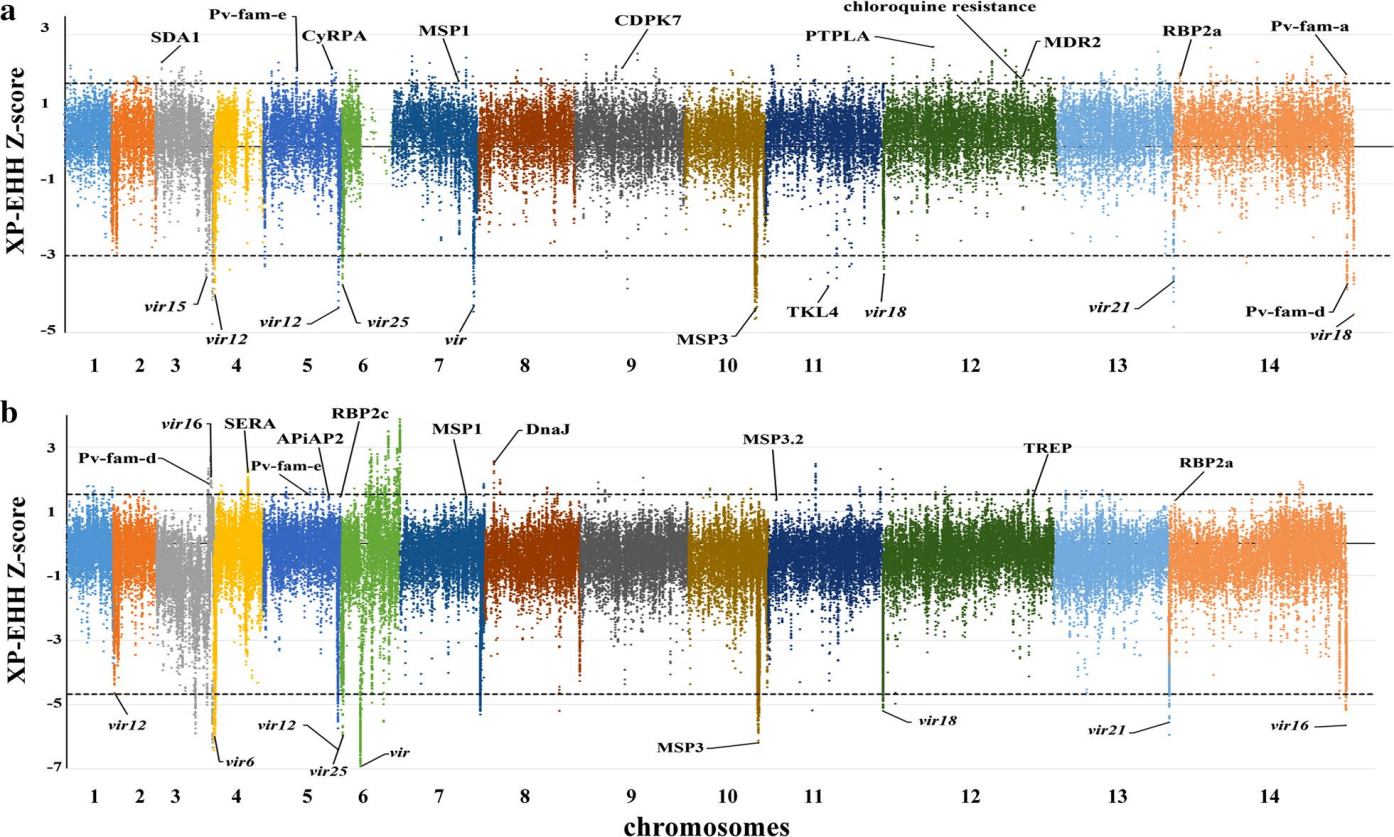
Genome-wide scan of standardized XP-EHH for *P. vivax* SNPs with minor allele frequency of at least 5% in CMB area. Individual chromosomes are identified by alternate colouring of their SNPs, with high scoring SNPs highlighted, indicating loci most likely to have been under recent positive directional selection. **a** Using 19 isolates obtained from different continents across the globe as the reference population. *Dashed lines* indicate the top 1% of XP-EHH values (±0.5%, XP-EHH score >1.7 or <−2.93). **b** Using 18 isolates from eight Asian countries as the reference population: Cambodia, Malaysia, Thailand, Vietnam, China, Myanmar, Laos, and Indonesia. *Dashed lines* indicate the top 1% of XP-EHH values (±0.5%, XP-EHH score >1.54 or <−4.68)

As a supplementary approach, XP-EHH test was applied in this study to compare the average haplotype length associated with each SNP between CMB samples and the references from other regions. It identifies areas in the genome where destination samples show much longer haplotypes than the reference, indicative of recent positive selection on the tested population. Some SNP loci were discarded because the physical distance (kbp) between two markers >200 and 80,024 loci were retained. As earlier indicated, the top 1% locus (±0.5%, XP-EHH score >1.7 or <−2.93) were concerned. These SNP loci includes 173 genes, 99 of them were annotated clearly (see Additional file 1: Table S2 for a complete list of the top 1% score genes). Some important gene encoding proteins associated with red blood cell invasion and immune evasion were observed in positive XP-EHH scores list (top 0.5%), such as RBP2a, MSP1P, MSP3, CyRPA and Pv-fam-a (Fig. 3a). The positive selection signals suggest that *P. vivax* in CMB area are facing more pressure to survive than in any other region. This led to higher ratios of diversity in the genes that are associated with host-parasite interactions. However, the selection signals that are close to some drug resistance genes were less effective when the reference was changed to Asian strains (Fig. 3b). Some genes encoding multidrug resistance protein 2 (MDR2) and chloroquine resistance marker (CRT) were absent on the top list of comparison between CMB and other Asian countries. This suggests that fast evolution in drug resistance genes is a common feature throughout the Southeast Asia region, where the recent selection of genes (*dhfr* and *dhps*) resistance to pyrimethamine and sulfadoxine have already been observed [8]. However, when the negative value list of XP-EHH was checked, *msp3* and *vir* families were found in the top list of both comparisons. The result suggests that the CMB samples do not possess such advantageous allele in these gene families, even though the variants were high in the population.

Previous studies have claimed that drug resistance is largely driven by positive selection in *Plasmodium*, and the XP-EHH test can be used to identify areas in the genome where resistant parasites show much longer haplotypes than sensitive parasites, indicating recent positive selection on the resistant population [13]. This study found positive selection signals in 11 drug resistance genes (see Additional file 1: Table S3), including *crt* (|iHS| = 7.28), *mdr2* (|iHS| = 5.37) and bifunctional dihydrofolate reductase-thymidylate synthase gene (*dhrf*-*ts*, |iHS| = 5.01) (Table 2). Their higher XP-EHH values indicate that the sweep is young and the signal has not yet been lost through recombination. As earlier noted, the severe changes in XP-EHH values of some drug resistance genes show that the fast evolution is not restricted to CMB area but ubiquitous throughout the Southeast Asia region. In *P. falciparum*, genes that produce the most abundant proteins in sporozoites have been investigated as vaccine candidates [34, 35]. Also, some of the candidates including circumsporozoite-related antigen and sporozoite invasion-associated protein were found to appear concurrently with higher iHS and XP-EHH scores.

### Genetic diversity demonstrate the selective sweep events in CMB samples

**Fig. 4:**
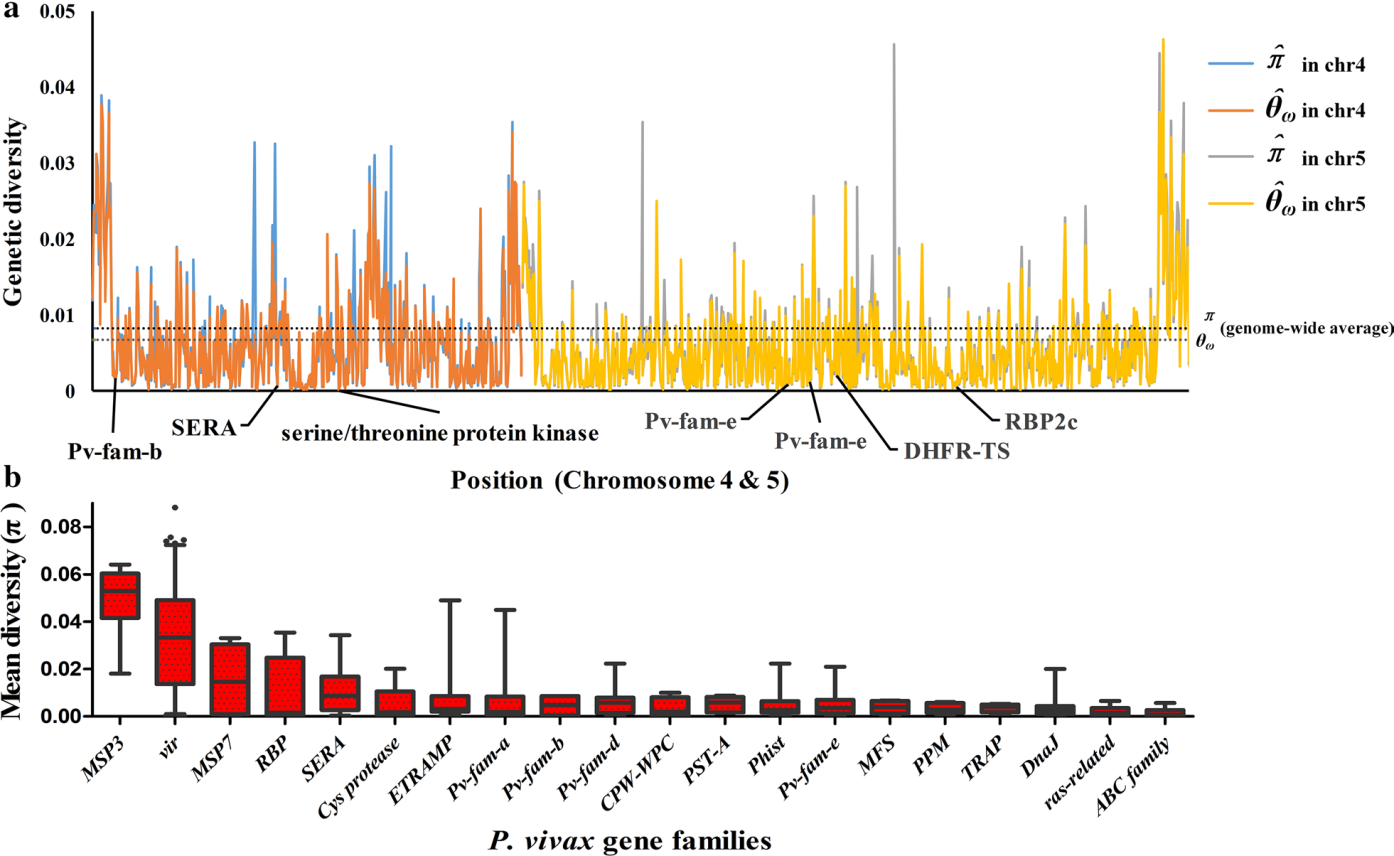
Diversity of *P. vivax* gene families and examples of selective sweep around some important genes in Chromosome 4 and 5. **a** Example of genetic diversity (*y-axis*) calculated in slide windows across Chromosome 4 and 5 (positions in *x-axis*). *Dashed lines* represent the genome-wide average of each diversity measure. **b** Mean pair-wise SNP diversity in *P. vivax* gene families. Gene families associated with merozoite invasion or immune response modulation show the highest diversity. *Red bars* on the *box plots* represent the 25th–75th percentile range, and *dots* indicate outlier genes

The nucleotide diversity ($\hat{\pi }$) and Watterson’s estimator ($\hat{\theta }$_ω_) were calculated to find the genome regions with unusually high or low genetic diversity in CMB area. On genome scale, $\hat{\pi }$ was estimated to be 0.0082 and $\hat{\theta }$_ω_ to be 0.0067, and genetic diversity is lower in exonic regions but higher in intronic and intergenic regions (Fig. 4a). Mean pair-wise divergence among the sequenced *P. vivax* isolates is higher in gene families encoding protein associated with red blood cell invasion and immune evasion (e.g. MSP3, VIR, MSP7, RBP, SERA) (Fig. 4b). On those regions which associated with the invasion genes, numerous genes were found at the centre of the low diversity region. The phenomenon in which functional mutations is surrounded by low diversity is the classic sign of hard selective sweep and accompanying beneficial alleles are rapidly raised. Selective sweep events were checked in the genes that were already chosen in iHS or XP-EHH calculation, and extended homozygosity regions with extremely low polymorphic SNPs were observed.

Lastly, higher XP-EHH or |iHS| scores were found on most of those genes which involved in drug resistance in *P. vivax*, especially the chloroquine resistance marker (PVX_118062) and *mdr2* (PVX_118100) (Table 2). These genes are known to confer resistance to chloroquine and pyrimethamine. The ongoing positive selective process suggests that the malaria control programme in Myanmar imposed huge pressure on *P. vivax* in CMB area and play an important role in the process of diversification.

## Discussion

### Genetic diversity in CMB area

Sequencing *P. vivax* genome gave us insights into the parasites’ biology but this has also raised some challenging questions. *P. vivax* diversity is affected by sampling size, previous work [36] showed the complex geographical pattern of *P. vivax* variation in Asia where human migration increases local genetic diversity and the Americas due to repeated introduction of *P. vivax* from many European countries. This study report a genome-wide survey of *P. vivax* isolates in CMB area, using blood samples from local patients with malaria to identify population-specific selective processes.

The result of this study showed that the genetic diversity estimated from CMB *P. vivax* isolates ($\hat{\theta }$_ω_ = 0.0067) is higher than the global sample of *P. falciparum* (where $\hat{\theta }$_ω_ is estimated to be 1.03 × 10^−3^ using isolates from Africa, America, Asia and Oceania) [37]. However, it is still difficult to compare the genetic diversity value to other *P. vivax* populations, although it is well known that *P. vivax* is more genetically diverse and less structured than *P. falciparum* [38]. For example, the $\hat{\theta }$_ω_ of *P. vivax* in Colombia population is estimated to be 7.0 × 10^−4^ [32] and even less than *P. falciparum*. Another study using 5.6 kb of non-coding DNA from *P. vivax* isolates across India reported $\hat{\theta }$_ω_ values ranging (from 1.3 × 10^−3^ to 3 × 10^−3^) [39]. These values are still lower than the diversity estimates from whole genome of *P. vivax* isolates from CMB area. Exploring the profile of variation in individual genes and gene families to evaluate the potential functional consequences of the extremely high genomic diversity, it showed that the mean pair-wise divergence among the CMB isolates is highest in gene families associated with red blood cell invasion and immune evasion [21, 40]. The top five families with diversity are genes encoding MSP3, VIR, MSP7, SERA and RBP, which correlates with previous studies [18]. It was reported in a previous study [41] that the genetic diversity in MSP3 ranges from 0.033 (MSP3H) to 0.088 (MSP3E) in Thailand, which is close to the results from CMB isolates (0.018–0.064). The result of this study exhibited large differences in this family, which allow the parasite to invade host cells. Furthermore, studies have reported that the genetic diversity in MSP7 family ranges from 0.0004 (MSP7F) to 0.039 (MSP7E) in Colombia [42]. Compared with result from this study with CMB isolates which ranges from (0.0004 to 0.033), there were close disparity. Study done by [31] found that very few reads could map 130 kb region at the subtelomeric end of chromosome 7. This is as a result of the sharp decline of the GC content along the subtelomeric region and the accompany enrichment of repeated sequences. The presence of artifacts is predicted due to poor coverage in regions with low complexity and this is typical of the *vir*, *sera* and *msp3* gene families. The early de-novo study with one of these samples (PV113) confirmed that independent deletion event exists but it does not appear in the CMB samples [43]. In conclusion, the CMB isolates show greater genetic diversity than isolates from other parts of the world at whole genome level. This evidence is consistent with their intense transmission level.

### Signatures of positive selection

Malaria transmission intensity and parasite genetic diversity are known to vary greatly in different parts of Southeast Asia due to variation in rainfall abundance and seasonality [44]. Since, positive selection is a type of natural selection where environment factors apply constant pressure over generations in favor of specific beneficial trait. Then, it is advantageous to apply selective sweep analyses at population level to study CMB populations because the parasite has a high rate of recombination and gene flow throughout the region. This study has identified parasite loci evidently under distinct processes of selection in a highly endemic population and the positive selection that cause the allele frequency to shift over time mainly in the gene families (Table 2). There are 18 *vir*, 4 *msp* and 6 *ApiAP2* genes in the top 1% iHS list (|iHS| > 5.93). The *sera* and *rbp* genes are also in the top list, with more of their paralog genes in the 5% list. Few genes appeared in the XP-EHH selection candidate list when compared with samples from other continents, but occurred only in individuals, such as: *msp1p* (PVX_099975), *rbp2a* (PVX_121920), *CyRPA* (PVX_090240) and *Pv-fam-a* (PVX_101515). Previous studies have shown different candidate genes under selection using different approaches. Hupalo et al. [7] explored the genomic profile of divergence and found some loci associated with drug resistance. Hupalo et al. findings are similar to the drug resistance candidates in iHS and XP-EHH test such as *crt* (PVX_087980) and *PI3K* (PVX_080480). Strong signals of selection on notable genes were also found in McDonald–Kreitman test, including those associated with red blood cell invasion and with the potentials of adapting to different regions in the host. Also, their list is very close to the prediction of the haplotype-based selection models. These include *ama1* (PVX_092275), *sera3* (PVX_003840), *msp7* (PVX_082650), *sera5* (PVX_003830), *maebl* (PVX_092975) and *Pv*-*fam*-*a* (PVX_092990). More so, using different comparative analysis study [45], Cornejo et al. identified 38 annotated genes with positive selection signature. Despite the application of different methodology, some of the results obtained from their findings were consistent with this study. It includes genes encoding DnaJ (PVX_084650) and helicase (PVX_088190).

Meanwhile, some gene families with high iHS value like *sera*, *PST*-*A* and *Phist* were totally absent in XP-EHH candidates. Findings show that 8 out of 11 genes in the MSP3 gene family have higher iHS value (top 5%) but lower negative XP-EHH value (bottom 0.5%). The evolution of *msp3* should be driven by multi-allelic diversifying selection and this will provide functional redundancy in terms of increasing antigenic diversity [46]. This suggests that the genetic diversity of *msp3* depends on sample size, and the CMB isolates would show negative XP-EHH value when compared on larger regional basis. Previous studies show that *P. vivax* strengthens the invasion and immune evasion capacities to meet the environmental stressors in demand [47]. Finding from this study is sympathetic to this view and exhibits even stronger local characteristics.

### Drug resistance of *Plasmodium vivax* in the China–Myanmar border area

In a recent study of *P. falciparum* in Southeast Asia, positive selection analysis using haplotype-based method identified loci involved in resistance to chloroquine (*crt*) in Thailand, Cambodia and Gambia, sulfadoxine–pyrimethamine (*dhfr* and *dhps*) in Cambodia, and artemisinin (*kelch13*) in Cambodia [34]. Decay of these signatures following changes in drug use appears to be rapid, facilitated by the high recombination rate of *Plasmodium*. In Myanmar, primaquine is used for radical treatment of *P. vivax* since 1951. Single dose of primaquine is used as gametocidal medicine for *P. falciparum* since 2002, and artemisinin-based combination therapy (ACT) is free of charge for all ages in public sector. More specifically, the chloroquine and primaquine combination therapy was used for vivax malaria in the China–Myanmar border area in recent years. The results obtained from the application of both iHS and the XP-EHH tests in this study, provide evidence of subtle differences in local selection signatures in the *P. vivax* isolates from CMB area that are likely to represent ongoing or very recent selection events. This pattern of low diversity that surrounds a fixed substitution is the classic sign of a selective sweep, in which the mutant allele is rapidly fixed by selection. Among the 11 drug resistance genes with positive selection signals, six genes are involved in chloroquine resistance, and three other genes are associated with multidrug resistance. The dihydrofolate reductase gene which confer resistance to pyrimethamine (*dhfr*-*ts*, PVX_089950) was also found [48]. A recent study in Peru found haplotypes in drug-resistance genes including *dhfr* and *mdr*, suggesting that resistance mutations have arisen independently and might be directly linked to the widespread use of chloroquine, artemisinin and primaquine [49]. Similarly, an abundance of chloroquine and multidrug resistance genes under strong positive selection should be considered as an inevitable consequence of enormous use of drug in Myanmar, where the drugs of choice are still chloroquine and primaquine combination therapy [50].

### *Plasmodium vivax* isolates from CMB area exhibit stronger regional features

Assessing and characterizing the genetic diversity of parasites known to constitute public health burden is important because it provides the platform to assess the direct effects of diversity on clinical disease and also generates reliable information that will help improve therapeutic efficacy and further enhance effective vaccine development [14]. These results further add to the growing evidence that *P. vivax* populations are genetically diverse. The genetic diversity of *P. vivax* obtained in this study is similar to findings in other (continents) where they are more transmitted. Also, this study has buttressed previous reports that *P. vivax* originated from Asia and that human migration increases local diversity [51]. The extreme diversity indicate the persistent synergy and prolonged association of *P. vivax* with humans in CMB area and this might pose serious challenge to the effective control of malaria cases in the area.

Furthermore, the census of genomic diversity in CMB *P. vivax* isolates shows high degree of genetic polymorphism, this may translate into important functional variation. The investigation of the evidence for selective sweeps due to drug pressure or other mechanisms was done and exhibited a number of genes with strong signatures of positive selection. It was observed that most of them are associated with red blood cell invasion and immune evasion. Also, some relatively low diversity genes such as *Pv*-*fam*-*e* (PVX_089475), *DnaJ* (PVX_092765) and *PST*-*A* (PVX_118700) also showed positive selection. This suggests that the Indoor-residual-spraying (IRS), larval control plan and treatment complexity subjects CMB’s *P. vivax* to more pressure for survival.

## Conclusion

This study assessed the genetic diversity of *P. vivax* genome sequence in CMB area to provide information on the positive selection of gene loci involved in red blood cell invasion and immune evasion, as well as drug resistance. It has also given insight into the genetic basis of drug resistance which can be applied to examine genomes using haplotype-based selection detecting methods. The signs of hard selective sweep involved in drug resistance genes were consistent with the history of drug use during national malaria elimination programme in this area, and they have also been identified. Identification of these signatures of positive selection allows for monitoring the emergence of drug resistance in parasite populations.

## Additional file

**Additional file 1: Table S1.** 485 genes with top 5% integrated haplotype score (|iHS| > 4.78). **Table S2.** 173 genes with top 1% (±0.5%) cross-population extended haplotype homozygosity value. **Table S3.** 11 drug resistance genes with high |iHS| and/or XP-EHH value and sweep feature.
